# Comparison and analysis of the biomechanics of the lower limbs of female tennis players of different levels in foot-up serve

**DOI:** 10.3389/fphys.2023.1125240

**Published:** 2023-02-24

**Authors:** Zhiqiang Liang, Jinan Wu, Jiabin Yu, Shanshan Ying, Zhiyong Liu, Yu Zhang, Yaodong Gu, Jianshe Li

**Affiliations:** ^1^ Faculty of Sports Science, Ningbo University, Ningbo, China; ^2^ Research Academy of Grand Health, Ningbo University, Ningbo, China; ^3^ School of Exercise and Health, Shanghai University of Sport, Shanghai, China

**Keywords:** foot-up serve, women’s tennis, biomechanics, motion control, lower limbs

## Abstract

**Purpose:** The purpose of this study was to examine biomechanical performance of the foot-up serve (FUS) in female tennis players at different skill levels.

**Methods:** FUS analysis was completed in the biomechanical laboratory by 32 female college tennis players at three different levels. During FUS, 3D-biomechanical data from tennis players’ lower limbs were collected. One-way ANOVA was used to examine differences in kinematic and kinetic data between groups

**Results:** Range of motion (ROM) of bilateral lower-limb joints revealed significant differences in kinematics performance during both the preparation and landing cushion phases (*p* < 0.05). During preparation, Level 3 was significantly longer than Level 2 (P-a = 0.042, P-b = 0.001, and P-c = 0.006). During the flight phase, significant differences between levels 1 and 3 (P-a:0.002) and levels 1 and 2 (P-c:0.000) were discovered (P-a:0.002 and P-c:0.000). There were significant height differences between levels 1 and 2 as well as between levels 1 and 3. (P-a = 0.001, P-c = 0.000). During serve preparation (P-c = 0.001) and landing, GRF’s peak was significantly higher than level 3. (P-c:0.007). Significant differences were found between groups in the LLS preparation stage, with level 3 significantly higher than levels 1 and 2. (P-a = 0.000, P-b = 0.001, and P-c = 0.000); during landing, level 2 LLS was significantly higher than levels 1 and 3. (P-a = 0.000, P-b = 0.000, and P-c = 0.035).

**Conclusion:** The range of motion of joints and the stiffness of the lower limbs have a significant impact on a tennis player’s FUS performance. A larger of joint mobility and lower-limb stiffness promote better performance during the FUS preparation stage.

## 1 Introduction

To compete at the highest levels in tennis, athletes must possess excellent speed, agility, muscle strength, and other abilities (Jaime et al., 2018). Athletes smash and serve regularly during tennis events in which serve is vital (Roetert et al., 2009). Thus, a powerful serve can easily win the match (Kovacs and Ellenbecker, 2011). Previous research has indicated that the tennis serve is closely related to the upper limbs, but there have been little investigations on the lower limbs (Buckley and Kerwin, 1988; Elliott et al., 1995; Elliott et al., 2003). The lower limbs provide the foundation of the tennis service chain. Lower-limb movement is essential for energy generation and transmission to the trunk, upper limbs, and racket (Kibler, 1995).

Tennis players’ lower limbs have a positive effect on how well their serves perform. Kovacs and Ellenbecker, (2011) claim that the tennis serve is a challenging motion that calls for athletes to coordinate their upper and lower limbs and joints in order to gather all forces generated on the ground and transfer them to the ball. Studies have shown a strong correlation between tennis serve speed and leg strength (Kaya et al., 2018). Reid et al. (2012) found that powerful performance of lower limbs can improve the racket’s capacity to support speed. Olivier et al. (2005) also discovered that whereas tennis players at different levels of competition all demonstrate the same explosive force when serving, their vertical ground reaction force and coordination of lower limb connections greatly varies. These variations in biomechanical traits could affect how well athletes perform in competition. Therefore, it is crucial to conduct research on the lower limbs of the tennis serve to enhance performance.

Tennis serves are classified into two types based on the position of the feet when serving: foot-up serve (FUS) and foot-back serve (FBS). FBS supports both feet stably, which provides high balance and accurate service, but the force is relatively weak (Sun et al., 2012; Filippo et al., 2016). FUS is completed by relying on the support of the front leg and stepping up with the back leg. FUS has a higher hitting point and greater strength than FBS because of the forward movement of the center of gravity, although its balance is relatively low (Filippo et al., 2016; Liang et al., 2017). The majority of tennis players prefer to use FUS during a match. As a result, using biomechanical analysis technology to analyze the lower limbs during FUS serving not only improves understanding of the mechanism of tennis athletes’ lower limbs but also has practical implications for tennis service training.

Here, the purpose of this study is to investigate lower-limb biomechanical performance in tennis players at multiple levels of performance during FUS using biomechanical analysis. China has combined athletic and academic endeavors to produce backup players for competitive sports since the 1980’s (Guo et al., 2022), and the findings of this study will be useful for the training of both athletic and academic female tennis players. Based on previous research, we hypothesize that lower limb biomechanical performance differs significantly due to diverse level of female tennis players.

## 2 Research methods

A cross-sectional study with three parallel groups was used in this paper. Through comparing biomechanical indicators between three diverse level of female tennis players, we would like to figure out which biomechanical indicators would differ due to players’ level.

### 2.1 Participants

A total of 32 female college tennis players participated in the test, and seven of them withdrew from the test due to test time, competition, and professional course conflicts. Finally, 25 female college tennis players, including seven national level 1 athletes (height: 178.0 ± 3.15 cm, weight: 58.5 ± 6.36 kg, and BMI: 18.46 ± 2.20 kg m^-2^), eight national level 2 athletes (height: 174 ± 2.10 cm, weight: 56 ± 2.83 kg, and BMI: 18.06 ± 1.20 kg m^-2^), and 10 students majored in the tennis special class (height: 168.5 ± 1.10 cm, weight: 54 ± 1.70 kg, and BMI: 19.01 ± 0.11 kg m^-2^), participated in the biomechanical test for FUS. Criteria for subject recruitment were as follows: 1) the right hand of subject was the dominant hand; 2) subject had received professional tennis training for at least 1 year; 3) lower limbs and feet had no injuries or diseases in the past 6 months; and 4) participants had no other factors that could affect completing FUS.

### 2.2 Biomechanical tests

#### 2.2.1 Biomechanical records

FUS test was performed out on the test participants in a laboratory. The kinematic and kinetic performance of the bilateral lower limbs during FUS was recorded using a 3D force platform (AMTI, United States of America) and an infrared Vicon motion capture system (Oxford Metrics Ltd, Oxford, UK). For kinematics, the frequency was 250 Hz, and for ground reaction force, it was 1000 Hz. Lower-limb stiffness (LLS), take-off height H), landing time (LT), ground reaction force (GRF), and lower-limb displacement (LLD) were the biomechanical indicators that were gathered. All data had been exported automatically by Vicon software.

#### 2.2.2 Joint anatomical position

**FIGURE 1:**
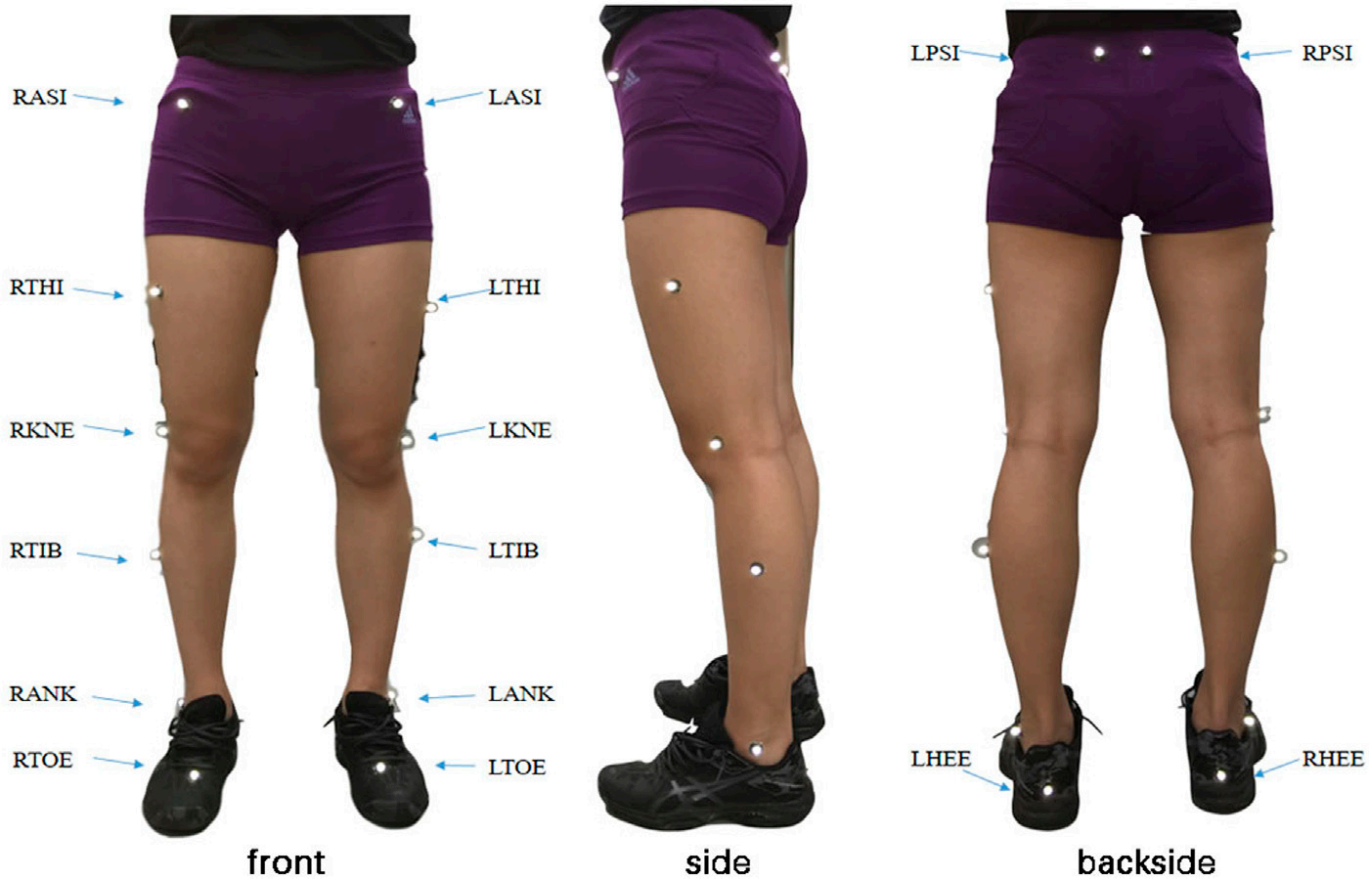
Lower-limb anatomy location in participants.

The kinematic data of both lower limbs of FUS were gathered in this study using the plug-in gait lower limb model (Figure 1). The experimental operators calibrated the athletes once they became accustomed to the experimental setting and constructed the three - dimensional image before the experiment. Then, using a ruler, the scientist completed the work required for personalized static modeling and measured the athletes’ bilateral lower limbs’ leg length, knee width, and ankle width. The lower limb joints of the athletes were then calibrated using reflecting markers by a scientist. The plug-in gait had 16 anatomical positions, including the left anterior superior spine (LASI), the right anterior superior spine (RASI), the left posterior superior spine (LPSI), the right posterior superior spine (RPSI), the left and right knees, the left and right tibias, the left and right ankles, the left and right toes, and the left and right heels (RTOE).

#### 2.2.3 Action division of FUS

Tennis’ service action could be divided into three stages: take-off before service, leaving, and landing cushioning, according to the literature (Harriss and Atkinson, 2009). In this study, the kinematics and dynamics of athletes’ takeoff and landing cushions were primarily examined.

### 2.3 Experimental protocol

**FIGURE 2:**
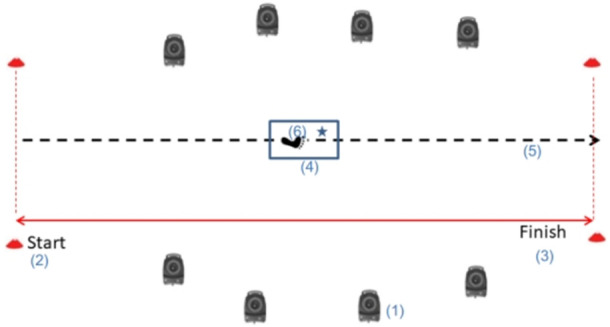
Experimental environment of FUS’s biomechanical test.

Three steps made up the experimental test process: calibration prior to the experiment and familiarization of the athletes with the testing environment; formal experimentation; and data collecting and analysis following the experiment. The athletes had enough time to get used to the experimental test environment before the test. Following that, participants successfully completed six FUS with flat shot (Figure 2) on the force platform while maintaining consistency and coordination in their movements. During FUS, the athletes’ bilateral lower limbs’ hip, knee, and ankle joints’ kinematic and kinetic data were gathered using a motion capture system. All participants were given 30 s to rest after each FUS to prevent fatigue. Throughout each test, the air conditioner maintained the test environment’s room temperature at roughly 26 C. Following the test, the examiner standardized the subject’s test results, and the participants engaged in relaxation exercises.

### 2.4 Data processing and analysis

#### 2.4.1 Take-off height

The athletes’ take-off height was calculated by using the take-off time, and the calculation formula was as follows: $height=\frac{\left(g\times ∆t^{2}\right)}{8}$ , g = 9.8 m/s, where ∆t is the take-off time of athletes.

#### 2.4.2 Calculation of LLS

FUS is mainly completed in the sagittal plane and by flexion of the knee joint. The hip joint and trunk remain relatively stable. Therefore, the LLS of athletes in the take-off preparation stage and landing r stage of FUS could be expressed by vertical stiffness. The calculation formula was as follows: $Kvert=\frac{Fmax}{∆y}$ ; where Fmax is the peak value of GRF, and ∆y is the vertical displacement of the body’s center of gravity. The vertical displacement could be calculated by the proportion of human morphological links (David, 2009), height, and angle of knee flexion extension ROM in the service preparation stage and the landing buffer stage. The calculation formula was as follows: $∆y=L_{thigh}-L\times \sqrt{{L_{shank}}^{2}+{L_{thigh}}^{2}-2\times L_{shank}\times L_{thigh}\times \cos\alpha }$ , $L_{knee}=0.039\times H$ , $L_{shank}=0.285\times H$ , $L_{thigh}=0.53\times H$ .

#### 2.4.3 Statistical analysis

The data were present as mean ± standard deviation (M±SD). Following the initial completion of standardization, the normal distribution of ROM, H, TOT, FT, LT, LLS, GRF, and LLD was tested (IBM, United States of America). One-way ANOVA was used to examine the differences between groups once the normal distribution test was finished. LSD *post hoc* analysis was used to analyze intergroup differences. P was set to 0.05. All analysis was performed using SPSS (IBM SPSS17.0, SPSS Inc).

## 3 Results

### 3.1 Kinematic results

#### 3.1.1 ROM of joints in FUS

**FIGURE 3:**
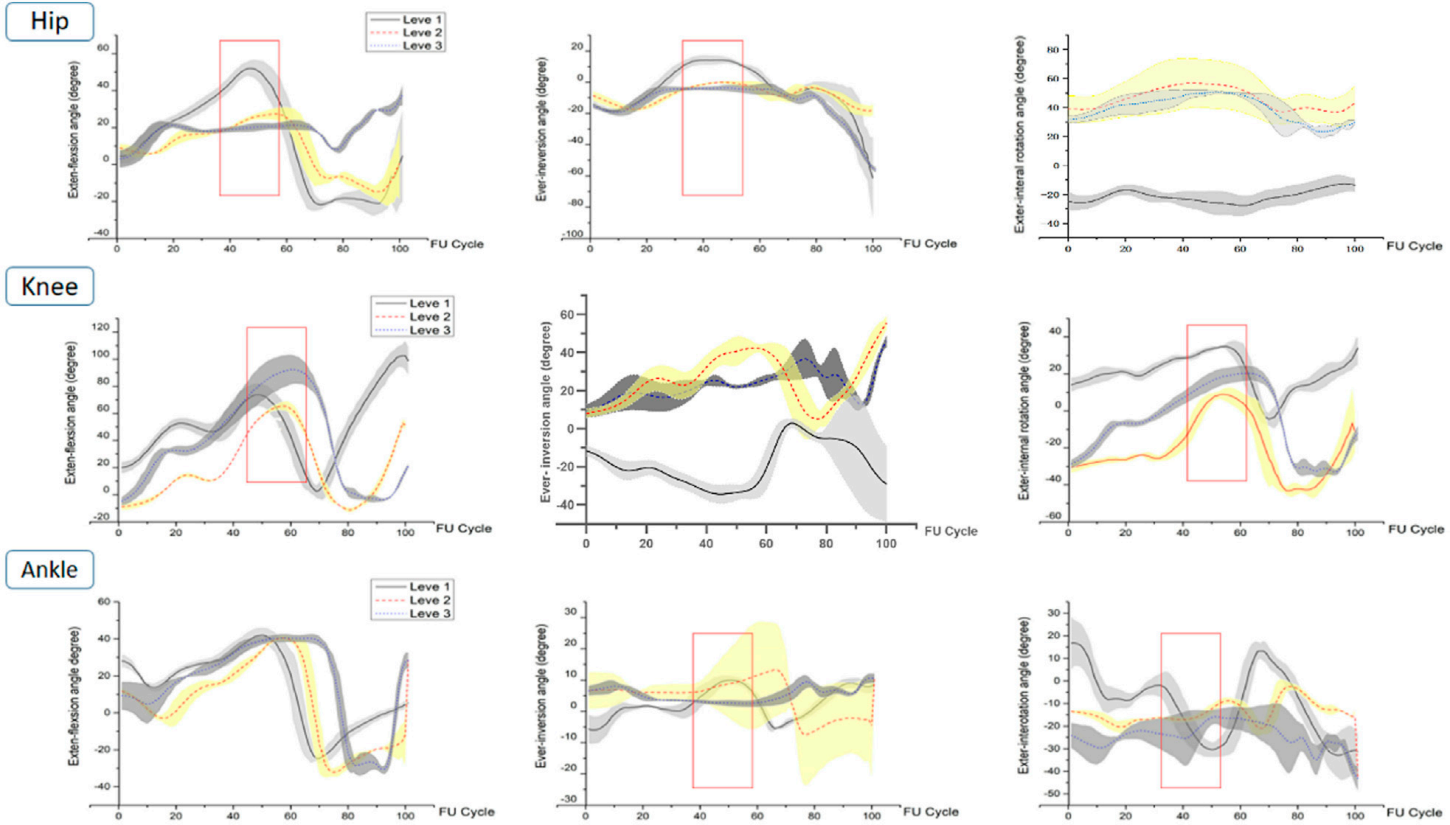
Right lower-limb joint ROM of level 1, level 2, and level 3 during FUS. The red rectangles represent the areas of difference in joint ROM during the take-off phase.

**FIGURE 4:**
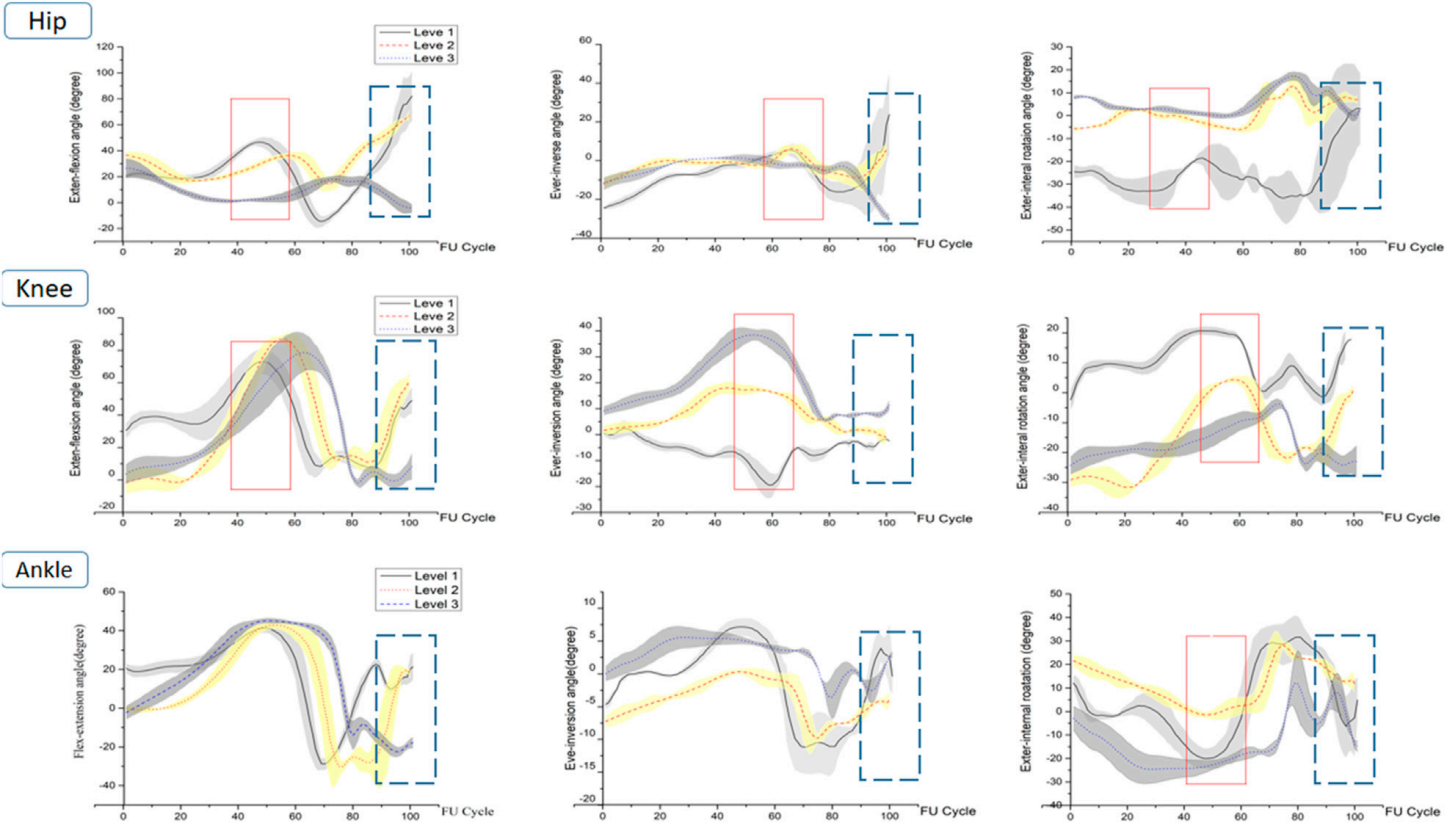
Left lower-limb joint ROM of level 1, level 2, and level 3 during FUS. (1) The red rectangles represent the areas of difference in joint ROM during the take-off preparation phase. (2) The blue dashed rectangles represent the areas of difference in joint ROM during the landing buffer phase.

Joint ROM performance of the bilateral lower-limb hip, knee, and ankle joints among groups during FUS preparation stage are shown in Figures 3, 4 and Table 1. Differences in the right lower-limb joints among three groups were found in hip and ankle joints. The flexion-extension, adduction-abduction, and internal rotation-external rotation ROMs of the hip were significantly greater in the level 1 and level 2 than in level 3 (P-a<0.001, P-b<0.001, and P-c = 0.001). The flexion-extension ROM in right lower-limb knee of level 1 was greater than the level 2 and level 3, however only ROM in knee of level 1 and level 2 was significantly different (P-a:0.002). Knee internal-external rotation ROM of Level 1 was significantly higher than level 3 (P-c:0.016). The differences of ROM in ankle in the coronal and horizontal planes showed similar trends (level 1 > level 3 > level 2) (P-a:<0.001, P-c:<0.001).

**TABLE 1:** Bilateral lower-limb joint ROM of level 1, level 2, and level 3 during the takeoff phase.

	Level	Right lower-limb				Left lower-limb				
Joint		ROM (deg)		*p*-value		ROM (deg)				*p*-value
		M	SD	P	M		SD	P		
Hip joint	level 1	77.27^ac^	2.46	a:0.000	55.30^ac^		6.60		a:0.000	
Flexion - extension	level 2	35.89^ab^	3.03	b:0.000	25.71^a^		2.20		c:0.000	
	level 3	22.95^bc^	4.01	c:0.001	29.81^c^		4.97		a:0.000	
	level 1	78.51ac	14.06	a:0.000	29.72^ac^		3.41		b:0.014	
Adduction - abduction	level 2	17.97^a^	1.59	c:0.000	19.20 ^ab^		2.53		c:0.000	
	level 3	17.12^c^	3.24	a:0.001	13.48 ^bc^		1.89		a:0.000	
	level 1	39.85^ac^	3.74	c:0.002	17.61		0.70		b:0.019	
Internal rotation - external rotation	level 2	28.62^a^	2.21	a:0.002	17.17		2.64		c:0.000	
	level 3	28.33c	3.55	b:0.001	17.95		2.67		a:0.048	
Knee joint	level 1	106.74^a^	7.31	c:0.001	61.14^ac^		3.15		b:0.000	
Flexion - extension	level 2	88.14	5.95	c:0.016	93.31^ab^		2.16		c:0.001	
	level 3	97.36	10.79	a:0.000	77.66^bc^		12.86		a:0.00	
	level 1	43.28^c^	2.47	b:0.043	22.97^ac^		4.82		b:0.00	
Adduction - abduction	level 2	43.42^b^	4.97	c:0.000	17.45^ab^		2.55		a:0.03	
	level 3	31.58^c^	3.55	a:0.000	34.30^bc^		2.26		b:0.02	
	level 1	44.59	5.12	c:0.000	21.59^ab^		3.33			
Internal rotation - external rotation	level 2	49.09	3.75		37.19^b^		1.73			
	level 3	52.73	3.75		20.02		4.07			
ankle joint	level 1	68.32	4.11		54.84		11.76			
Metatarsus flexion - dorsiflexion	level 2	71.24	4.35		67.21		16.34			
	level 3	62.99	10.34		63.55		1.75			
	level 1	18.54^ac^	3.00		15.45		4.06			
Inversion - eversion	level 2	4.80^a^	1.12		10.55		1.64			
	level 3	8.20^c^	1.52		9.92		4.20			
	level 1	57.57^ac^	10.59		43.61^a^		7.85			
Internal rotation - external rotation	level 2	17.66^a^	4.89		30.78^b^		3.79			
	level 3	26.54^c^	5.44		48.17		9.12			

During the preparation for FUS, significant differences in the hip and knee joints of the left lower limb were found (Figure 4; Table 2). Hip flexion-extension ROM was substantially larger at level 1 than at levels 2 and 3. Hip adduction-abduction ROM significantly increased with athletes’ level (P-a:<0.001, P-b:0.014, P-c: <0.001). Knee flexion-extension ROM showed a level 2 > level 3 > level 1 trend across groups, however there was a significant difference between them. In terms of the ROM for knee internal-external rotation, level 2 was followed by levels 1 and 3. However there were significant differences between levels 1 and 2, Levels 2 and 3. Ankle internal-external ROM across levels 1 and 2, as well as between levels 2 and 3, differed significantly.

**TABLE 2:** Left lower-limb joint ROM of level 1, level 2, and level 3 during the landing phase.

	Level	Left lower-limb					
Joint		ROM (deg)			*p*-value		
		M	SD			P	
Hip joint	level 1	46.86^ac^		14.03			a:0.004
Flexion - extension	level 2	20.60^a^		1.84			c:0.000
	level 3	6.07c		2.23			a:0.000
	level 1	38.31^ac^		3.54			b:0.034
Adduction - abduction	level 2	16.48^ab^		1.17			c:0.000
	level 3	10.92^bc^		2.96			a:0.027
	level 1	18.98^ac^		8.96			c:0.009
Internal rotation - external rotation	level 2	7.18^a^		4.10			b:0.000
	level 3	4.54^c^		1.18			c:0.002
Knee joint	level 1	36.60^b^		4.86			a:0.000
Flexion - extension	level 2	48.67^c^		10.54			b:0.000
	level 3	11.19^bc^		4.77			b:0.000
	level 1	2.90^a^		1.49			c:0.000
Adduction - abduction	level 2	5.02^c^		1.65			a:0.000
	level 3	3.74		2.05			b:0.000
	level 1	19.05^b^		5.98			a:0.000
Internal rotation - external rotation	level 2	21.13^c^		4.00			c:0.004
	level 3	3.09^bc^		0.70			a:0.003
Ankle joint	level 1	11.43^a^		2.37			
Metatarsus flexion - dorsiflexion	level 2	49.15^ab^		6.99			
	level 3	6.04^b^		0.16			
	level 1	11.28^ac^		0.88			
Inversion - eversion	level 2	2.35^a^		1.79			
	level 3	5.11^b^		2.43			
	level 1	33.54^a^		2.34			
Internal rotation - external rotation	level 2	8.88^a^		4.33			
	level 3	21.41		10.05			

^a^ Represented a significant difference between level 1 and level 2, *p* < 0.05.

^c^ Represented a significant difference between level 2 and level 3, *p* < 0.05.

^b^ Represented a significant difference between level 3 and level 1, *p* < 0.05.

The hip joint’s ROM during landing matched that of the FUS preparation stage, which was level 1 > level 2 > level 3. There were no differences between levels 2 and 3 in terms of internal-external rotation ROM or flexion-extension ROM, though. Similar trends were observed in the knee’s flexion-extension ROM and internal-external rotation ROM; level 2 was significantly greater than level 1 and level 3, respectively (P-b:0.019, P-c:0.002; P-b:0.000, P-c:0.000). Level 2 had a significantly larger adduction-abduction ROM of the knee than the other levels. Ankle flexion-extension ROM was considerably higher at levels 1 and 2 compared to level 3 with level 2 being higher than level 1. However, only the inversion-eversion ROM between level 1 and level 2, between level 2 and level 3, and the internal-external rotation ROM between levels 1 and 2 showed significant differences. The trend of ankle inversion-eversion ROM and internal-external rotation ROM among the three groups was level 1 > level 3 > level 2.

#### 3.1.2 Time and flying height in FUS’s different phases

**FIGURE 5:**
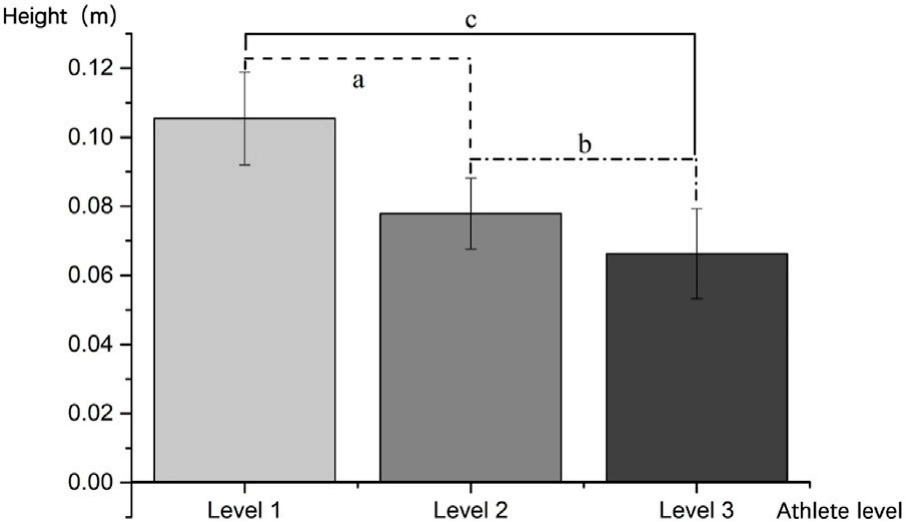
FUS’s flying height of level 1, level 2, and level 3.

The amount of time level 1, level 2, and level 3 participants took to complete FUS at various points varied significantly (Table 3). Level 3 was significantly longer than Level 2 during preparation (P-a = 0.042, P-b = 0.001, and P-c = 0.006). However, during flight phase, only the differences between level 1 and level 3 (P-a:0.002) and level 1 and level 2 (P-c:0.000) were significantly found (P-a:0.002 and P-c:0.000). LT of level 2 was much higher during landing than that of level 3 (P-b:0.035). Details of the FUS-H are shown in Table 4 and Figure 5 for levels 1, 2, and 3. Significant differences between level 1 and level 2 and between level 1 and level 3 were found in groups where the trend of H was level 1 > level 2 > level 3. (P-a:0.001, P-b:0.125, and P-c:0.000).

**TABLE 3:** Time spent in FUS’s different phases of level 1, level 2, and level 3.

Phase	Level	Time (s)		*p*-value
		M	SD	
TOT				
	Level 1	0.90^ac^	0.07	a:0.042
	Level 2	1.16^ab^	0.07	b:0.000
	Level 3	1.55^bc^	0.37	c:0.006
FT				
	Level 1	0.29^ac^	0.02	a:0.002
	Level 2	0.25^a^	0.02	
	Level 3	0.23^b^	0.02	c:0.000
LT				
	Level 1	0.14	0.01	
	Level 2	0.16^c^	0.06	b:0.035
	Level 3	0.12^c^	0.02	

^a^ Represented a significant difference between level 1 and level 2, *p* < 0.05.

^b^ Represented a significant difference between level 3 and level 1, *p* < 0.05.

^c^ Represented a significant difference between level 2 and level 3, *p* < 0.05.

**TABLE 4:** FUS’s flying height of level 1, level 2, and level 3.

Level	FT(s)	H(m)	*p*-Value
Level 1	0.29 ± 0.02	0.11 ± 0.01^ac^	a:0.001
Level 2	0.25 ± 0.02	0.08 ± 0.01^a^	b:0.125
Level 3	0.23 ± 0.02	0.07 ± 0.01^c^	c:0.000

^a^ Represented a significant difference between level 1 and level 2, *p* < 0.05.

^b^ Represented a significant difference between level 2 and level 3, *p* < 0.05.

^c^ Represented a significant difference between level 3 and level 1, *p* < 0.05.

### 3.2 FUS-GRF

**FIGURE 6:**
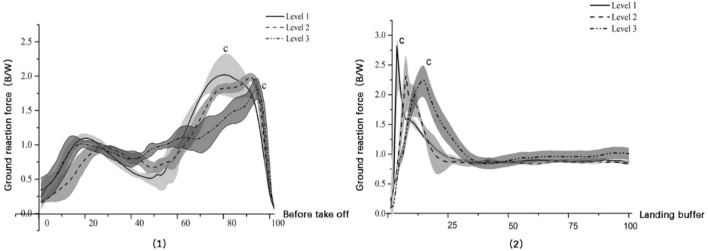
FUS’s GRF variations in take-off (Right) and landing (Left) phases.

GRFs among three groups showed a bimodal peak force during serve preparation (Figure 6; Table 5). GRF`s peak of level 1 and level 2 were slightly higher than level 3; only significant difference between level 1 and level 3 was found (P-c:0.001). During landing, GRF peak of level 1 were significantly higher than level 3 (P-c:0.007), but there was no significant difference between level 1 and level 2 and between level 2 and level 3. The time of the GRF peak significantly moved forward with the increase in athlete level. (P-a:0.000 and P-c:0.000).

**TABLE 5:** Timing characteristic of GRF in level 1, level 2, and level 3 during takeoff and landing phases.

Level	GRF- peak value (B/W)	*p*-value	Stage of emergence (%)	*p*-value
Before take-off				
Level 1	2.02 ± 0.32^c^	a:0.117	76.61 ± 2.68^ac^	a:0.000
Level 2	1.99 ± 0.22	b:0.056	90.39 ± 0.82^a^	b:0.075
Level 3	1.81 ± 0.32^c^	c:0.001	92.65 ± 1.49^c^	c:0.000
Landing phases				
Level 1	2.81 ± 0.33^c^	a:0.127	2.29 ± 0.51^ab^	a:0.000
Level 2	2.31 ± 0.55	b:0.152	6.54 ± 1.19^bc^	b:0.000
Level 3	2.30 ± 0.19^c^	c:0.007	11.44 ± 1.34^ac^	c:0.000

^a^ Represented a significant difference between level 1 and level 2, *p* < 0.05.

^b^ Represented a significant difference between level 2 and level 3, *p* < 0.05.

^c^ Represented a significant difference between level 3 and level 1, *p* < 0.05.

### 3.3 FUS-LLS

The differences in LLS during the landing and FUS preparation are shown in Table 6. Significant differences were seen in the preparation stage of LLS between groups, with level 3 significantly higher than level 1 and level 2 (leve3>level l > level 2). (P-a:0.000, P-b:0.001, and P-c:0.000). However, during landing, LLS of level 2 was much higher than that of levels 1 and 3. (P-a:0.000, P-b:0.000, and P-c:0.035).

**TABLE 6:** Lower-limb stiffness of level 1, level 2, and level 3 during FUS.

Level	GRF- peak value (B/W)	LLD (cm)	LLS	P
Before take-off				
Level 1	2.02 ± 0.32	34.7 ± 1.50	5.81 ± 3.76^ab^	a:0.000
Level 2	1.99 ± 0.22	64.2 ± 0.29	3.09 ± 0.65^bc^	b:0.001
Level 3	1.81 ± 0.32	16.87 ± 0.67	10.92 ± 0.36^ac^	c:0.000
Landing phases				
Level 1	2.81 ± 0.33	45.22 ± 0.32	6.25 ± 0.72^ab^	a:0.000
Level 2	2.31 ± 0.55	25.52 ± 0.31	9.80 ± 1.19^bc^	b:0.000
Level 3	2.30 ± 0.19	30.64 ± 0.19	7.42 ± 0.62^ac^	c:0.035

^a^ represented a significant difference between level 1 and level 2, *p* < 0.05.

^b^ Represented a significant difference between level 2 and level 3, *p* < 0.05.

^c^ Represented a significant difference between level 3 and level 1, *p* < 0.05.

## 4 Discussion

FUS has the advantage of hitting height and speed when compared to FBS. As a result, a thorough biomechanical analysis of FUS will help athletes improve their athletic performance. We compared the biomechanical performance of both lower limbs during FUS at levels 1, 2, and 3. Results showed that those who performed at a higher level had greater ROM throughout the FUS preparation stage but significantly different ROM in the coronal and horizontal planes of the knee and ankle, according to the results. The significant large joint ROM was used to land, but different levels of lower-limb joints take on different cushioning responsibilities during landing. Higher level participants demonstrated faster serve preparation times, longer take-off times, and more body displacement than lower level participants. The peak force point of GRF advanced gradually as athletes’ performance improved during the preparation and landing phases. While FUS preparation improved subject performance, LLS demonstrated significantly different subject performance during the landing cushion. Different biomechanical performance across various performance levels during FUS point to the existence of a different dynamic movement chain, which would result in a different muscle working model and increased risk of injury and disease. Further research into the mechanisms and consequences behind this biomechanical performance is deserves.

### 4.1 Kinematic performance of FUS

Tennis players need to practice in frequent and consistent special training in order to achieve high-level sports performance and skilled movement abilities (Ben Kibler and Safran, 2000). Even young tennis players at a lower level require specialized training for an average of 6.1 days per week and 2.3 h per day to be in good competitive condition (Kibler et al., 1988). Tennis movement skills, which are crucial for the formation and development of tennis athletes’ movement abilities and the prevention of sports injuries, can be improved with the help of biomechanical theories and methods, which can be used by coaches, tennis players, and scientists (Elliott, 2006). Therefore, to enhance tennis performance and avoid injuries, biomechanical research techniques are required to understand the process of each stroke.

In this study, the ROM of bilateral lower limb joints of participants with higher performance level were greater than that of participants with lower performance level, especially the ROM of the hip joint of the left leg and the ROM of the hip joint, knee joint, and ankle joint of the right leg. As seen by previous studies, tennis players with a large ROM can better activate muscles during sports, allowing them to achieve greater take-off strength (Hu, 2009). Tennis service requires coordination as well as explosive acceleration ability of trunk and lower limb muscles (Liu, 2008). Higher levels of lower-limb strength and efficiency during take-off serve can provide a foundation for athletes’ serve accuracy and lower-limb and trunk explosive force from movement technology (Yan et al., 2000; Jin and Qu, 2008). Therefore, the athletes’ lower limb extensor muscle groups’ eccentric contraction time and work distance were extended due to the large ROM of the joint prior to hitting the ball, allowing them to conserve a greater amount of elastic potential energy at this point. This larger ROM of the joint during the preparation stage of FUS is more conducive to the athletes’ serve. However, an excessively large ROM may raise the burden of the lower limb extensors during training, which is unfavorable to the lower-limb takeoff during service. Additionally, it would have an impact on the coordination of the lower limb connections and the angular velocity of joints, which would have an impact on how well muscles store elastic potential energy and produce force.

During tennis serve, participants perform the landing by changing the ROM of joints. However, this ROM motion is not an independent activity, and it is accompanied with changes in ROM of adjacent joints (Chun-Man et al., 2011). Our results are supporting this finding. We also found that participants mainly relied on the left lower limb for landing cushion. Level 1 was conducted through ROM of hip and ankle joints, level 2 was conducted through ROM of the knee joint, and level 3 was relatively insignificant conducted through lower-limb ROM. These results are in line with research of Chun-Man et al. (2011) and Blackburn and Pauda (2008), which tennis player cushion the impact of landing by increasing the ROM of lower limb joints. These suggest that tennis service mainly depends on the athletes’ single lower limb to perform the landing, the athletes’ single lower limb needs to buffer or transfer the energy generated during the takeoff preparation of both lower limbs. This cushion strategy during landing is similar to the landing cushioning mechanism of volleyball athletes, which may lead player into a high risk of injury (Boling and Padua, 2013).

Studies on the control of lower limbs upon landing are critical for avoiding sports injuries. According to research, there is a clear relationship between the function of the hip and knee joints and the changes in the lower limb mechanism during landing (Boling and Padua, 2013). Power (2010) investigated the relationship between abnormal mechanical hip joint performance and knee joint injury and discovered that weak hip muscle force increases the degree of flexion of the ipsilateral trunk at the landing stage and increases the load of knee joint abduction. The increased muscle strength of the hip muscle group not only improves control of the athletes’ lower limb movements during the landing phase and reduces the load on the knee and ankle joints during the landing phase, but it also supports the athletes’ trunk stability during the landing phase. As a result, we proposed that athletes be encouraged to use the muscles surrounding the hip joint to cushion the landing.

### 4.2 Spatial performance and GRF of FUS

FUS may be separated into three stages: preparation, flight, and landing buffer based on the force distribution of the foot during serve. When preparing for takeoff, TOT of individuals with greater athletic levels was shorter than that of their competitors. Higher exercise levels were associated with longer FT and higher H during the flight phase, whereas lower performance levels were associated with the inverse. We also discovered that during the FUS preparation and landing cushion phases, the peak of GRF at level 1 was substantially larger than that at level 3. The force between foot and ground has been the source of FUS’s strength. The GRF peak value, peak time phase, and action time all had a significant impact on impulse change. As a result, the law of conservation of impulse and momentum could be applied. Classical mechanics defines impulse as a process that involves the accumulation of force and time on objects as well as the increase in momentum (Yang et al., 2014). It is typically impacted by variables like quality, peak force, timing, and instantaneous load rate. More momentum can be gained in sports by people with greater sports levels than by those with lower sports levels. We discovered that the acquisition of impulse and the accumulation of momentum of athletes during the preparation stage of FUS had a more significant impact on the performance of the subsequent takeoff phase based on the characteristics of GRF and the temporal and geographical parameters of FUS. As a result, the FUS preparation phase warrants special attention from coaches and sports scientists.

### 4.3 Lower-limb stiffness performance in FUS

LLS of tennis players is related to their explosive output ability, according to Durand et al. (2010), and the higher LLS level, the higher reverse jump height. In the triple jump, Rabita et al. (2008) discovered that the LLS level of triple jumpers with enhanced training experience is significantly higher than that of jumpers without such training experience. Hobara et al. (2010) investigated LLS in endurance athletes and found similar results to Rabita et al. (2008). The biomechanical study of tennis also found that the LLS of athletes have a special impact on the efficiency of foot movement and help athletes achieve higher sports performance (Durand et al., 2010). Appropriate LLS can stimulate the degree of muscle activation to obtain a higher level of GRF and motion impulse through ROM (Harriss and Atkinson, 2009). This experiment discovered that the LLS of level 3 was greater than that of levels 1 and 2. The higher LLS at level 3 was primarily due to a smaller ROM, which LLS could not compensate for in terms of FUS performance. Bouhlel et al. (2006) discovered that moderate intensity exercise training can significantly improve the LLS of young male athletes’ lower limbs. Hobara et al. (2008) discovered that different types of exercise can improve the LLS of participants’ lower limbs. FUS is an explosive action, so explosive and enhanced exercise may be appropriate for tennis players when combined with sport-specific characteristics (Harrison et al., 2004). LLS is linked to sports injuries. In general, too high LLS can easily cause bone tissue structure damage, whereas too low LLS can easily cause soft tissue structure damage (Butler et al., 2003). Grimston et al. (1991) and Radin et al. (1978) discovered that excessive peak values of force, exercise loading, and impact loading put athletes at risk of injury and bone tissue damage.

Therefore, athletes with higher levels GRF peaks in the serve preparation stage would result in a higher risk of injury than athletes with lower levels. In addition, we also found that LLS of level 2 and level 3 was much higher than that of level 1 in the landing cushion phase. The kinematic analysis results showed that those with higher sports level had larger ROM than those with lower sports level in the landing cushion phase. Zhang et al. (2000) and Devita and Skelly (1992) found that increased knee ROM of joint flexion and extension to reduce moment of joint in the landing cushion phase can lead to reduced joint stiffness. Therefore, when coaches discover that athletes use smaller ROM to land, they should timely limit the movement mode or prompt athletes to land by increasing the ROM of sagittal joints, so as to avoid the occurrence of sports injury caused by exorbitant LLS.

Kinematic and kinetic analyses supported the hypothesis of this study, and revealed significantly different lower extremity biomechanical performance in tennis players serving at various sport levels; thus, real-time monitoring of lower extremity biomechanical performance during serving would not only help to improve tennis serving performance, but would also provide athletes with a guarantee for reduced sports injury risk. However, the study has some limitations. For example, the study did not analyze surface electromyography (sEMG) in the experimental test; the analysis of joint working patterns during the serve may have some limitations; and it is necessary to include sEMG in the follow-up study to comprehensively understand the working patterns of lower limb muscles during the serve. Second, the study did not record the information of sports equipment in detail, such as shoes and racket types; this imperfect recording of may also have an impact on the understanding of the participants’ basic characteristics and movement characteristics; thus, in order to improve the in-depth understanding of the study results, the information of the participants’ equipment should be recorded in the follow-up study. The final limitation is the small sample size. Despite the fact that our study’s sample size is in line with previous studies, this small size may not provide adequate statistical power. I Increasing the sample size might provide more insights into understanding of the biomechanical outcomes of tennis serves in future studies.

## 5 Conclusion

Women tennis players with varying levels of performance exhibit significantly different lower-limb biomechanical performance. The ROM of joints and the stiffness of the lower limbs have a major impact on a tennis player’s FUS performance. Better performance during the FUS preparation stage is facilitated by a greater range of joint mobility and stiffer lower limbs. The lower-limb stiffness, however, has an impact on the tennis player’s risk of injury during the landing cushion. Therefore, we propose that real-time player monitoring is necessary to assist different level women tennis players in enhancing their FUS performance.

## Data Availability

The original contributions presented in the study are included in the article/supplementary material, further inquiries can be directed to the corresponding author.
